# Finasteride withdrawal induces anxiety‐like behavior and novelty avoidance in adult male rats

**DOI:** 10.1111/jne.70150

**Published:** 2026-02-27

**Authors:** Lucia Cioffi, Silvia Diviccaro, Gabriela Chrostek, Francesco Paolo Ulloa Severino, Silvia Giatti, Diego Scheggia, Roberto Cosimo Melcangi

**Affiliations:** ^1^ Department of Pharmacological and Biomolecular Sciences “Rodolfo Paoletti” Università degli Studi di Milano Milan Italy; ^2^ Cajal Institute, CSIC Madrid Spain

**Keywords:** anxiety‐like behavior, hyperactivity, novelty avoidance, post‐finasteride syndrome, side effects

## Abstract

Finasteride, an inhibitor of the enzyme 5alpha‐reductase, prescribed for benign prostatic hyperplasia and androgenetic alopecia, induces a wide variety of side effects during treatment and upon withdrawal, like sexual dysfunction and cognitive and psychological disorders, inducing the so‐called post‐finasteride syndrome (PFS). Here, we explored the behavioral effects of this drug in adult male rats after subchronic finasteride treatment (20 days) and at drug discontinuation (1 month). We employed multiple behavioral paradigms, including the open field test and elevated plus maze to assess locomotor activity and anxiety, and a novelty‐seeking test to evaluate exploratory behavior and approach‐avoidance tendencies. Our results revealed a dichotomy between immediate and delayed finasteride effects. While effects after subchronic treatment were mild, significant behavioral alterations emerged at the withdrawal. In particular, pronounced hyperactivity, decreased center exploration in the open field, and marked avoidance of novel stimuli, collectively indicating an anxiety‐like behavioral phenotype, were revealed. These results, showing a picture of increased vulnerability, are in agreement with clinical reports in PFS, highlighting the relevance of our model for this condition. Moreover, the data here described strengthen the importance of monitoring patients not only during treatment but also following discontinuation of finasteride therapy.

## 1 INTRODUCTION

Finasteride (i.e., Propecia or Proscar) is an inhibitor of the enzyme 5alpha‐reductase (5α‐R) clinically approved for the treatment of benign prostatic hyperplasia (BPH) and androgenetic alopecia (AGA).^1^, ^2^, ^3^ 5α‐R exerts a key role not only in the peripheral tissues but also in the activation of some neuroactive steroids (i.e., steroids synthesized from peripheral glands as well as directly in the nervous system and controlling nervous functions), such as testosterone (T) and progesterone (PROG).^4^ T and PROG are metabolized by 5α‐R into dihydrotestosterone and dihydroprogesterone, respectively. These neuroactive steroids are then further converted by the action of 3α‐hydroxysteroid oxidoreductase or 3β‐hydroxysteroid oxidoreductase into further metabolites, such as 5α‐androstane‐3α,17β‐diol or 5α‐androstane‐3β,17β‐diol in the case of dihydrotestosterone and allopregnanolone or isoallopregnanolone in the case of dihydroprogesterone. Neuroactive steroids are important physiological modulators of nervous function and possible therapeutic agents for neurodegenerative and psychiatric disorders.^5^, ^6^

The description of finasteride as a well‐tolerated and relatively safe drug has recently been challenged by experimental findings that led to a more critical re‐evaluation of these concepts. Indeed, this drug induces a wide variety of side effects during treatment and upon withdrawal, such as sexual dysfunction and cognitive and psychological disorders, hallmarks of the so‐called post‐finasteride syndrome (PFS).^2^, ^7^, ^8^, ^9^, ^10^, ^11^, ^12^, ^13^

For instance, regarding the psychological domain, a report collecting 131 web‐based surveys of patients lamenting symptoms at finasteride withdrawal^14^ indicated mental cloudiness or brain fog (75% of cases) and elevated anxiety (74% of cases). As also described in another similar study, the psychological and cognitive complaints reported from PFS patients were decreased self‐confidence, irritability or easily flying into a rage, nervousness, agitation, inner restlessness, depression, hopelessness, feelings of worthlessness, suicidal thoughts, anxiety, panic attacks, sleep problems, decreased initiative and difficulty in concentration, mental confusion, forgetfulness or loss of short‐term memory, losing train of thought or reasoning, slurred speech, or stumbling over words.^15^ Clinical studies have also confirmed depressive symptoms. For instance, using the PHQ‐9 depression scale, Beck Depression Inventory, and Hamilton Depression Scale 17^10^, ^16^ or K‐10, Mini‐International Neuropsychiatric Interview, and Beck Depression and Anxiety Inventory,^15^ the presence of DSM‐IV major depressive disorder was confirmed in patients with PFS. In addition, functional magnetic resonance imaging confirmed abnormalities in brain regions implicated in depression and sexual arousal, such as the nucleus accumbens and prefrontal cortex.^16^

Studies in adult male rat models of finasteride treatment and PFS show controversial results. For instance, signs of depression‐like phenotype using a forced swim test (FST) were observed after 1 month of withdrawal but not immediately after treatment in one case^17^ and immediately after the finasteride treatment in another.^18^ Recent studies have shown depression‐like behavior not only after finasteride treatment,^19^ but also after drug withdrawal.^20^ In addition, there is evidence that motivational behavior was also negatively affected by finasteride treatment.^18^ Finally, it was reported that finasteride treatment causes the insurgence of anxiety‐like behaviors.^19^

In order to further explore the behavioral effects of finasteride, we evaluated anxiety‐like behavior using the open‐field test and elevated plus maze, as well as novelty‐seeking behavior and exploratory drive using the novelty‐seeking test (NST). These parameters were evaluated after chronic finasteride treatment (20 days) and at drug discontinuation (1 month).

## 2 MATERIALS AND METHODS

### 2.1 Animals

Adult male *Sprague–Dawley* rats (200–225 g, 2 months old at arrival; Charles River Laboratories, Lecco, Italy) were housed in the Department of Pharmacological and Biomolecular Sciences, University of Milan, for behavioral testing. All procedures were conducted in compliance with national (Legislative Decree No. 26, March 4, 2014) and international guidelines (EU Directive 2010/63, September 22, 2010) and were approved by the local ethics committee and the Italian Ministry of Health (authorization no. 261‐2021‐PR). All animals were kept in standard rat cages with food and tap water available ad libitum and under controlled humidity and temperature. Animals were allowed to acclimate for 1 week before the start of the experimental procedures.

### 2.2 Treatment and experimental design

**FIGURE 1:**
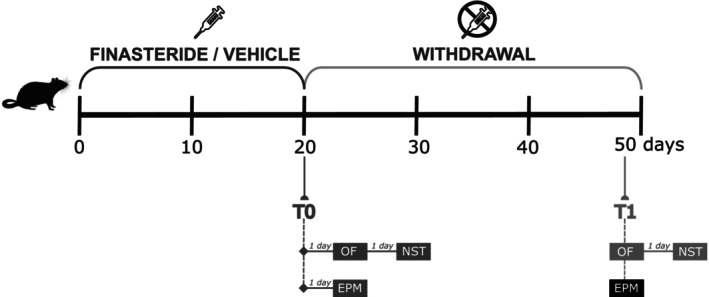
Schematic representation of experimental schedule and behavioral analyses performed. Open field (OF) and novelty seeking test (NST) were, respectively, evaluated 24/48 h after the last injection (T0) and 1 month after treatment suspension (T1) using batch 1 of animals. Elevated plus maze test (EPM) was evaluated 24 h after the last injection (T0) and 1 month after treatment suspension (T1) using batch 2 of animals.

Finasteride (3 mg/kg/day; Sigma‐Aldrich, Italy) was dissolved in corn oil containing 5% ethanol (v/v) and administered subcutaneously at a volume of 100 μL per day for 20 consecutive days. The finasteride dose here applied was based on our previous published observation.^17^, ^21^, ^22^, ^23^, ^24^ A total of 40 rats were used in the study, randomly assigned to two groups (20 animals per group): a vehicle‐treated group (vehicle) receiving the corn oil/ethanol solution alone, and a finasteride‐treated group (finasteride) receiving the drug in the same vehicle. Animals were randomly divided into two batches, and the experimental schedule and behavioral analyses performed are summarized in Figure 1. All animals were evaluated at two time points. Thus, 24/48 h after the last injection (T0) and 1 month after treatment cessation (T1). Open field and novelty‐seeking test were, respectively, performed using batch 1 of animals and elevated plus maze in batch 2.

### 2.3 Open field‐based behavioral assays

To assess anxiety‐like and novelty‐seeking behaviors, rats were tested at two separate sessions, in an open field arena (dimensions: 50 × 50 cm, walls 40 cm high) located in a well‐lit room. The animals were initially placed in the center of the arena and allowed to explore freely for 30 min. Behavior was monitored using a video tracking system (ANY‐maze, Stoelting), which recorded locomotor parameters including total distance traveled and time spent in predefined zones of the arena. Locomotor activity was quantified as the total distance traveled during the session, while anxiety‐like behavior was assessed based on the time spent in the center of the arena. In a separate session, the same arena was used to evaluate novelty‐seeking behavior by introducing two distinct objects differing in shape and color, placed in opposite corners. No familiarization phase was included, allowing for the assessment of spontaneous exploratory behavior in response to novel environmental stimuli. Time spent in close proximity to each object was recorded and used as an index of exploratory motivation, which can reflect emotional state and also anxiety‐related traits. The sessions described above were performed on two consecutive days. Additionally, animals were tested at two distinct time points (i.e., T0 and T1) to assess behavioral changes following subchronic finasteride treatment and at drug withdrawal, respectively.

### 2.4 Elevated plus maze behavioral assays

The elevated plus maze (EPM) apparatus consisted of two open arms (50 × 10 cm) and two closed arms (50 × 10 × 40 cm), arranged perpendicularly around a central square platform (10 × 10 cm) and elevated 50 cm above the floor. Each animal was placed at the center of the maze, oriented toward one of the open arms, and allowed to explore freely for a 5‐min period. Animal behavior, including movement and spatial position, was automatically tracked and quantified using ANY‐maze software. In a few cases we could not quantify behavior due to technical problems related to video tracking. Moreover, one animal of the vehicle group died a few days after arrival.

### 2.5 Statistical analysis

All statistical analyses were performed in GraphPad Prism (Version 7.2, La Jolla, CA) (version 7.2). Data were analyzed using a two‐way analysis of variance (ANOVA, including repeated measures), with *time* and *treatment* as two independent variables, followed by Tukey's multiple comparisons test. The normal distribution was assessed using the Kolmogorov–Smirnov test (*p* < 0.05 was considered significant) and outliers were identified and excluded based on Grubbs' test (α = 0.05). Statistical differences (in the two stages of OF and NST as well as in the session of EPM test) between vehicle and finasteride treated rats were assessed by Student's *t*‐test or Mann–Whitney *U* test for each behavioral parameter. Exact adjusted *p* values are listed in the figures for each experiment; where absent, the difference was not significant (*p* > 0.05).

## 3 RESULTS

### 3.1 Subchronic finasteride administration causes limited behavioral changes

**FIGURE 2:**
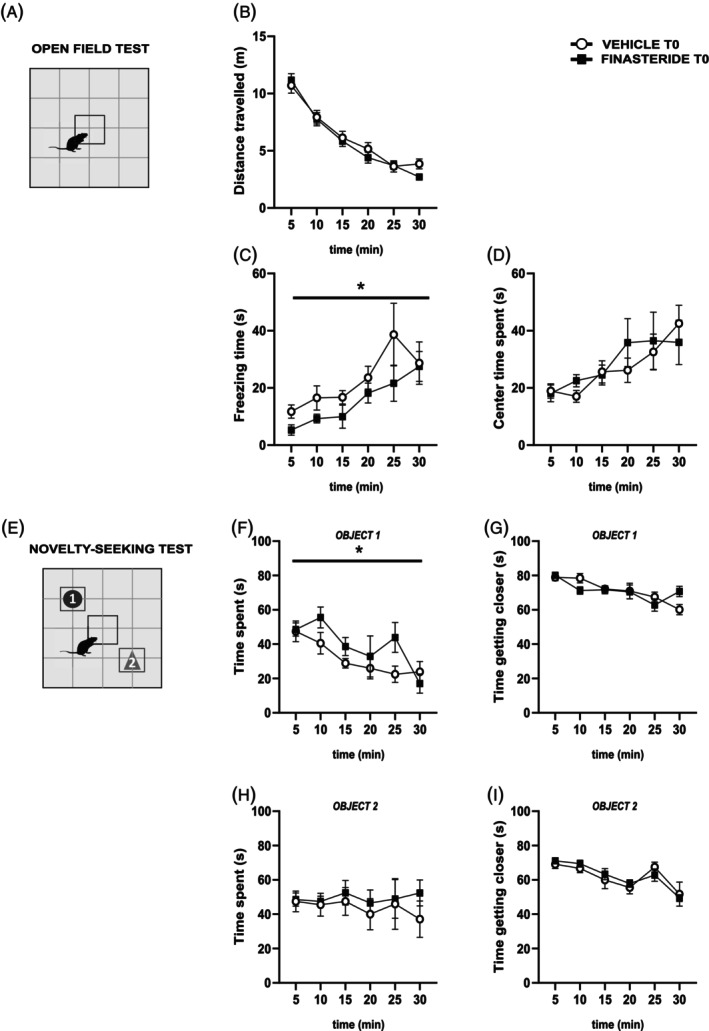
Effects of subchronic finasteride administration on behavioral parameters. (A) schematic representation of the open field arena (OF) test; (B) distance travelled by finasteride‐treated rats compared to control vehicle rats in the OF over 30 min (*n* = 10 (vehicle), *n* = 10 (finasteride)); (C) freezing time of finasteride‐treated rats compared to control vehicle rats in the OF over 30 min (*n* = 10 (vehicle), *n* = 10 (finasteride)); (D) center time spent by finasteride‐treated rats compared to control vehicle rats in the OF over 30 min (*n* = 10 (vehicle), *n* = 10 (finasteride)); (E) schematic representation of novelty‐seeking test (NST); (F) time spent approaching the object 1 by finasteride‐treated rats compared to control vehicle rats over 30 min (*n* = 10 (vehicle), *n* = 10 (finasteride)); (G) time spent to get closer to object 1 by finasteride‐treated rats compared to control vehicle rats over 30 min of the NST (*n* = 10 (vehicle), *n* = 10 (finasteride)); (H) time spent approaching the object 2 by finasteride‐treated rats compared to control vehicle rats over 30 min of the NST (*n* = 10 (vehicle), *n* = 10 (finasteride)); (I) time spent to get closer to object 2 by finasteride‐treated rats compared to control vehicle rats over 30 min of the NST (*n* = 10 (vehicle), *n* = 10 (finasteride)). *p < 0.05; values are expressed as mean ± SEM.

As a first step in evaluating the behavioral effects of finasteride treatment in adult male rats (Figure 2), we assessed locomotor activity and center time spent after 20 days of subchronic drug treatment (T0). As represented in panel A, we tested the rats in an open field arena and monitored their behavior over 30 min. Although we did not find a significant difference in overall locomotion, measured as total distance travelled (panel B, two‐way RM ANOVA, time × treatment, *F*_(5,108)_ = 0.65, *p* = 0.6620), we found that finasteride‐treated rats spent less time in freezing compared to controls (panel C, two‐way RM ANOVA, time × treatment, *F*_(5,108)_ = 0.51, *p* = 0.7667, treatment, *F*_(1,108)_ = 6.06, *p* = 0.0154). To assess potential anxiety‐like behavior, we analyzed the time spent in the center of the arena and found no differences between groups (panel D, two‐way RM ANOVA, time × treatment, *F*_(5,108)_ = 0.53, *p* = 0.7555).

In the second session, to investigate whether finasteride treatment affects novelty‐seeking behavior and exploratory drive, a two‐different object test was performed (Figure 2). In this setting, we presented animals with two objects that differ in shape and color to assess their natural exploratory behavior in the absence of the cognitive component of novelty recognition (panel E). This test specifically evaluates the motivation to explore new stimuli in the environment, which could be an important indicator of the emotional state and anxiety‐like behavior in rodents.

The analysis of exploratory patterns was performed separately for the two objects. In the case of object 1, we observed a reduction in the total exploration (panel F, two‐way RM ANOVA, time × treatment, *F*_(5,108)_ = 1.19, *p* = 0.3191, treatment, *F*_(1,108)_ = 4.41, *p* = 0.0380), but not in approach behaviors (panel G, two‐way RM ANOVA, time × treatment, *F*_(5,108)_ = 2.20, *p* = 0.0595). As for what concerns object 2, no differences were observed for any of the parameters analyzed (panels H, time spent approaching object 2, two‐way RM ANOVA, time × treatment, *F*_(5,108)_ = 0.19, *p* = 0.9671 and I, time spent to get closer to object 2, two‐way RM ANOVA, time × treatment, *F*_(5,108)_ = 0.43, *p* = 0.8269). These results suggest that subchronic finasteride treatment has limited behavioral effects.

### 3.2 Long‐term behavioral alterations following discontinuation of finasteride

**FIGURE 3:**
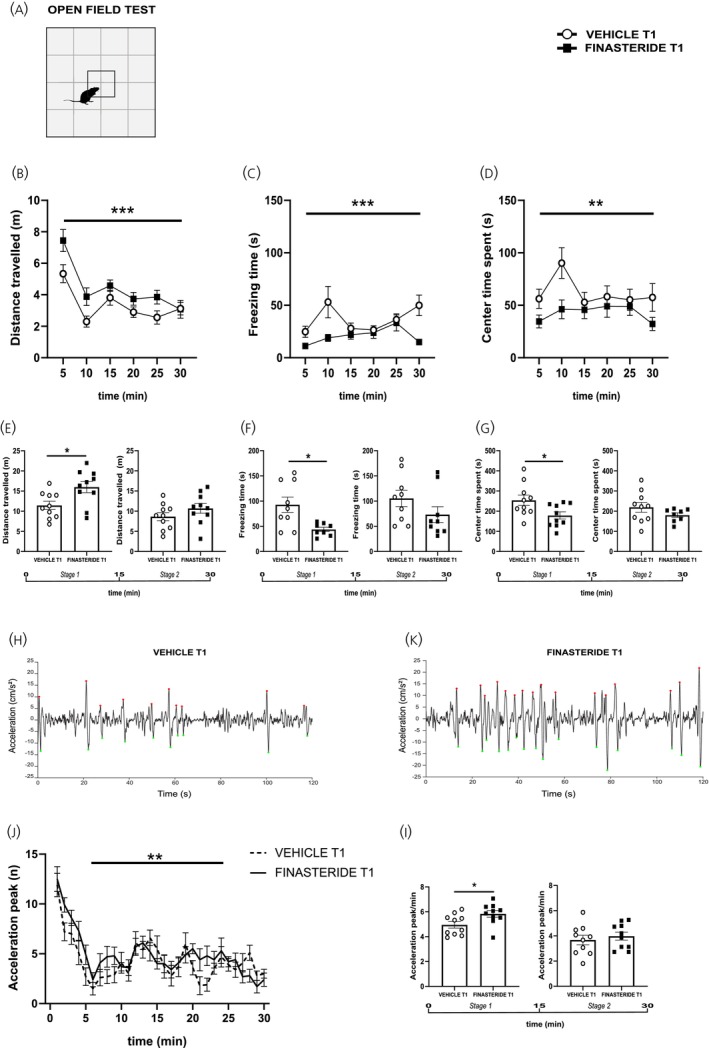
Effects of finasteride suspension on locomotor activity related to anxiety‐like behavioral state. (A) schematic representation of the open field arena (OF) test; (B) distance travelled by finasteride‐treated rats compared to control vehicle rats in the OF over 30 min (*n* = 10 (vehicle), *n* = 10 (finasteride)); (C) freezing time of finasteride‐treated rats compared to control vehicle rats in the OF over 30 min (*n* = 9 (vehicle), *n* = 8 (finasteride)); (D) center time spent by finasteride‐treated rats compared to control vehicle rats in the OF over 30 min (*n* = 9 (vehicle), *n* = 9 (finasteride)); (E) distance travelled by finasteride‐treated rats compared to control vehicle rats during the stage 1 (first 15 min) and stage 2 (second 15 min) in the OF (*n* = 10 (vehicle), *n* = 10 (finasteride)); (F) freezing time of finasteride‐treated rats compared to control vehicle rats in the OF during the stage 1 and stage 2 (*n* = 9 (vehicle), *n* = 8 (finasteride); *n* = 9 (vehicle), *n* = 9 (finasteride)); (G) time spent in the center by finasteride‐treated rats compared to control vehicle rats in the OF during the stage 1 and stage 2 (*n* = 10 (vehicle), *n* = 10 (finasteride)); (H and K) representative profile of acceleration vehicle and finasteride‐treated rats after 1 month drug discontinuation; (J) acceleration peak of finasteride‐treated rats compared to control vehicle rats over 30 min of OF test (*n* = 10 (vehicle), *n* = 10 (finasteride)); (I) frequency of acceleration of finasteride‐treated rats compared to control vehicle rats in the OF during the stage 1 and stage 2 (*n* = 10 (vehicle), *n* = 10 (finasteride)). *p < 0.05, **p < 0.01, ***p < 0.001. Values are expressed as mean ± SEM.

Next, as reported in Figure 3, we investigated the long‐term effects of subchronic finasteride treatment by testing the behavior of rats 30 days after discontinuation (T1) with the same approach as described above. At this time point, animals were re‐exposed to the open field arena (Figure 3A), and we found that finasteride‐treated rats exhibit significantly increased locomotor activity (panels B, total distance travelled, two‐way RM ANOVA, time × treatment, *F*_(5,108)_ = 1.21, *p* = 0.3086, treatment, *F*_(1,108)_ = 15.74, *p* = 0.0001), and markedly decreased freezing time (panels C, two‐way RM ANOVA, time × treatment, *F*_(5,102)_ = 2.24, *p* = 0.0564, treatment, *F*_(1,102)_ = 14.98, *p* = 0.0002) compared with controls. Moreover, these animals spent significantly less time in the center of the open field compared to controls (panel D, two‐way RM ANOVA, time × treatment, *F*_(5,106)_ = 1.09, *p* = 0.3705, treatment, *F*_(1,106)_ = 10.73, *p* = 0.0014). Because open‐field behavior exhibits robust within‐session habituation, with novelty‐driven exploration being most prominent during initial exposure, behavioral data were additionally examined in early (stage 1: 0–15 min) and late (stage 2: 15–30 min) phases. This analysis indicated a time‐dependent effect of finasteride withdrawal as all the above effects were statistically significant when analyzed after 15 min, during initial environmental exploration, but not in the late phase of testing (panels E, distance travelled, two‐tailed unpaired *t*‐test, *t* = 2.64, df = 18, *p* = 0.0165; *t* = 1.30, df = 18, *p* = 0.2098; F, freezing time, two‐tailed unpaired *t*‐test, *t* = 2.95, df = 15, *p* = 0.0099; two‐tailed Mann–Whitney test, *U* = 21, *p* = 0.0902 and G, time spent in the center, two‐tailed unpaired *t*‐test, *t* = 2.42, df = 18, *p* = 0.0265; *t* = 1.34, df = 16, *p* = 0.1988). The avoidance of exposed areas could be an indicator of increased anxiety‐like behavior. Because exploratory behavior consists of a rapid alternation between progressing and stopping, using the rats' trajectories, we then analyzed their movement patterns through the arena (panels h and k). We found that the finasteride‐treated rats at T1 showed a higher number of acceleration peaks compared with the control rats (panel J, acceleration peak, two‐way RM ANOVA, *F*_(29,540)_ = 1.32, *p* = 0.1246, treatment, *F*_(1,540)_ = 7.47, *p* = 0.0065), particularly during the first 15 min of the testing (panel I, frequency of acceleration, two‐tailed unpaired *t*‐test, *t* = 2.34, df = 18, *p* = 0.0307; *t* = 0.63, df = 18, *p* = 0.5387). These results are consistent with an increased anxiety‐like behavioral state, where animals exhibit more erratic or impulsive movement patterns.

**FIGURE 4:**
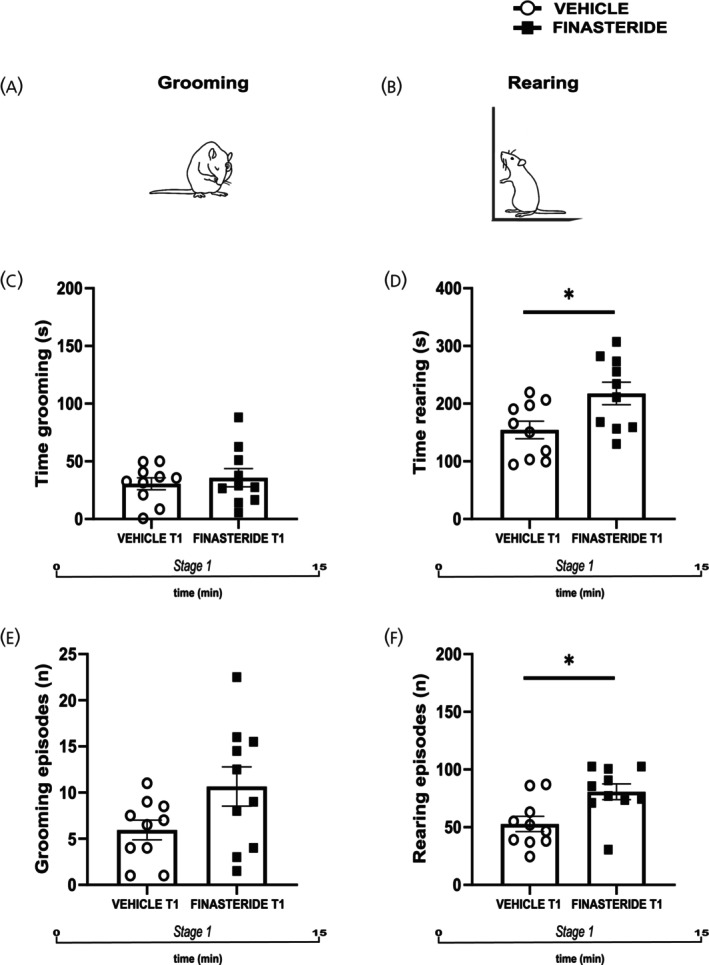
Effects of finasteride suspension on exploratory and self‐directed behaviors. (A) representation of grooming; (B) representation of rearing; (C) time spent in grooming during the stage 1 (*n* = 10 (vehicle), *n* = 10 (finasteride)); (D) time spent in rearing during the stage 1 (*n* = 10 (vehicle), *n* = 10 (finasteride)); (E) number of grooming episodes (*n* = 10 (vehicle), *n* = 10 (finasteride)); (F) number of rearing episodes (*n* = 10 (vehicle), *n* = 10 (finasteride)). *p < 0.05. Values are expressed as mean ± SEM.

To further explore these behavioral alterations, we analyzed grooming and rearing behaviors, as measures of self‐directed and exploratory activities, during the initial 15 min of the open field session at T1 (Figure 4A,B). Finasteride withdrawal did not significantly alter grooming time or the number of grooming episodes compared with vehicle‐treated animals (Figure 4C two‐tailed unpaired *t*‐test, *t* = 0.55, df = 18, *p* = 0.5885 and Figure 4E two‐tailed Mann–Whitney test, *U* = 28, *p* = 0.0923). Conversely, a significant increase in rearing time and number of rearing episodes was observed in finasteride‐treated rats relative to controls (Figure 4D, two‐tailed unpaired *t*‐test, *t* = 2.55, df = 18, *p* = 0.0199 and F, two‐tailed Mann–Whitney test, *U* = 19, *p* = 0.0181). This enhancement in vertical exploration confirms an elevated hyper‐reactivity, or arousal, state during the early phase of testing, consistent with the hyperlocomotor and anxiety‐like profile described above.

### 3.3 Finasteride discontinuation induces marked avoidance behaviors

**FIGURE 5:**
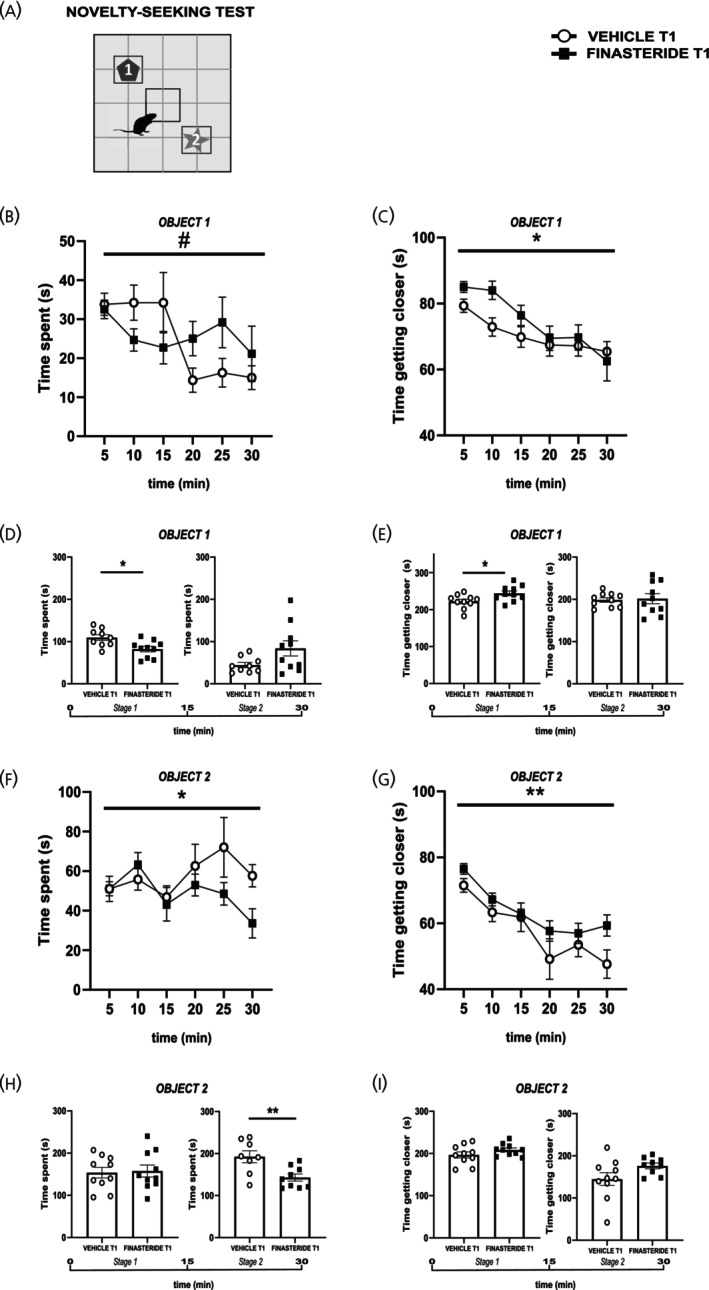
Effects of finasteride discontinuation on anxiety‐like behavior. (A) experimental timeline and schematic representation of novelty‐seeking test (NST); (B) time spent approaching the object 1 by finasteride‐treated rats compared to control vehicle rats after 1 month of drug discontinuation over 30 min of NST (*n* = 9 (vehicle), *n* = 9 (finasteride)); (C) time spent to get closer to object 1 over 30 min of NST (*n* = 9 (vehicle), *n* = 9 (finasteride)); (D) time spent approaching the object 1 in stage 1 (first 15 min) and stage 2 (second 15 min) of the NST (*n* = 9 (vehicle), *n* = 10 (finasteride); *n* = 9 (vehicle), *n* = 10 (finasteride)); (E) time spent to get closer to object 1 in stage 1 and stage 2 of the NST (*n* = 10 (vehicle), *n* = 10 (finasteride); *n* = 10 (vehicle), *n* = 10 (finasteride)); (F) time spent approaching the object 2 by finasteride‐treated rats compared to control vehicle rats after 1 month of drug discontinuation over 30 min of NST (*n* = 9 (vehicle), *n* = 10 (finasteride)); (G) time spent to get closer to object 2 over 30 min of NST (*n* = 9 (vehicle), *n* = 9 (finasteride)); (H) time spent approaching the object 2 in stage 1 (first 15 min) and stage 2 (second 15 min) of the NST (*n* = 10 (vehicle), *n* = 10 (finasteride); *n* = 8 (vehicle), *n* = 9 (finasteride)); (I) time spent to get closer to object 2 in stage 1 and stage 2 of the NST (*n* = 10 (vehicle), *n* = 10 (finasteride); *n* = 10 (vehicle), *n* = 9 (finasteride)). ^#^ *p* < 0.05 (time × treatment), *p < 0.05, **p < 0.01, ***p < 0.001. Values are expressed as mean ± SEM.

The finding that increased locomotion and avoidance of the center of the open field arena is statistically significant only during the first 15 min of the test (Figure 3E–G,I) suggested an anxiety‐like behavioral state related to the exposure to the novel environment. Thus, we challenged rats' tendency for novel stimuli exploration by introducing two other unfamiliar objects into the open field arena (Figure 5A) following the discontinuation of finasteride administration. At T1, control rats exhibited a sequential exploratory pattern, spending time investigating one object and then the other (panels B, two‐way RM ANOVA, time × treatment, *F*_(5,104)_ = 2.46, *p* = 0.0375 and F, two‐way RM ANOVA, time × treatment, *F*_(5,107)_ = 1.41, *p* = 0.2282, treatment, *F*_(1,107)_ = 4.02, *p* = 0.0474). In contrast, finasteride‐treated rats spent less time exploring both objects overall. This effect was particularly evident for one object during the first 15 min (panel D, two‐tailed unpaired *t*‐test, *t* = 2.89, df = 17, *p* = 0.0102; *t* = 2.00, df = 17, *p* = 0.0618), and for the other object in the following 15 min (panel H, two‐tailed unpaired *t*‐test, *t* = 0.21, df = 18, *p* = 0.8381; *t* = 3.10, df = 15, *p* = 0.0073), suggesting that the rats explored the objects in a time‐dependent sequence. Furthermore, we found that finasteride‐treated rats showed an increase in the time required to approach the objects (panels C, two‐way RM ANOVA, time × treatment, *F*_(5,106)_ = 1.03, *p* = 0.4018, treatment, *F*_(1,106)_ = 4.76, *p* = 0.0313 and G, two‐way RM ANOVA, time × treatment, *F*_(5,105)_ = 0.63, *p* = 0.6765, treatment, *F*_(1,105)_ = 8.02, *p* = 0.0055), in line with the anxiety‐like behavior patterns described above. In particular, we found a significant difference for one object in the first 15 min (panels E, two‐tailed unpaired *t*‐test, *t* = 2.45, df = 18, *p* = 0.0250; t = 0.23, df = 18, *p* = 0.8194), while for the other object (panel I, two‐tailed unpaired *t*‐test, *t* = 1.385, df = 18, *p* = 0.1831; *t* = 1.79, df = 17, *p* = 0.0911) we found no specific temporal effect.

**FIGURE 6:**
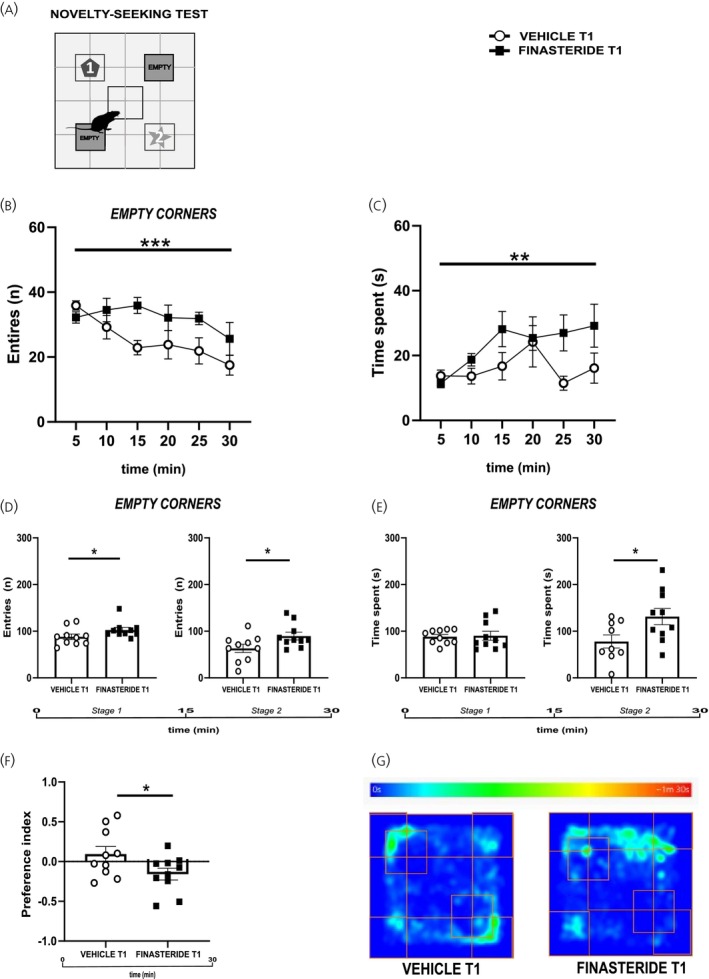
Effects of finasteride discontinuation on avoidance behavior. (A) Schematic representation of novelty‐seeking test (NST) and empty corners in the arena; (B) numbers of entries in the empty corners by finasteride‐treated rats compared to control vehicle rats after 1 month of drug discontinuation over 30 min of NST (*n* = 10 (vehicle), *n* = 10 (finasteride)); (C) time spent in the empty corners over 30 min of test by vehicle‐ and finasteride‐treated rats (*n* = 8 (vehicle), *n* = 9 (finasteride)); (D) numbers of entries in the stage 1 (first 15 min) and stage 2 (second 15 min) of the NST (*n* = 10 (vehicle), *n* = 10 (finasteride); *n* = 10 (vehicle), *n* = 10 (finasteride)); (E) time spent in the empty corners by vehicle‐ and finasteride‐treated rats in the stage 1 (first 15 min) and stage 2 (second 15 min) of the NST (*n* = 10 (vehicle), *n* = 10 (finasteride); *n* = 9 (vehicle); *n* = 10 (finasteride)); (F) preference index of time spent approaching the objects over 30 min of object‐test by finasteride‐treated rats compared to control vehicle (*n* = 10 (vehicle), *n* = 10 (finasteride)); (G) representative heat‐map of the time spent by vehicle‐ and finasteride‐treated rats in the arena. *p < 0.05, **p < 0.01, ***p < 0.001. Values are expressed as mean ± SEM.

We then analyzed the time spent in the zones without objects (Figure 6A) and found that finasteride‐treated rats displayed an increased number of entries throughout the test (panel B, two‐way RM ANOVA, time × treatment, *F*_(5,108)_ = 1.50, *p* = 0.1967, treatment, *F*_(1,108)_ = 12.70, *p* = 0.0005 and panel D, two‐tailed Mann–Whitney test, *U* = 22, *p* = 0.0336; *U* = 24, *p* = 0.0500) and a consistent increased time spent in these zones (panel C, two‐way RM ANOVA, time × treatment, *F*_(5,104)_ = 1.19, *p* = 0.3170, treatment, *F*_(1,104)_ = 7.84, *p* = 0.0061), which is significant during the last 15 min (panel E, two‐tailed unpaired *t*‐test, *t* = 0.19, df = 18, *p* = 0.8500; *t* = 2.34, df = 17, *p* = 0.0315), compared to control animals. Finally, to quantify this behavioral pattern, we computed a *preference index*, where negative scores indicate object avoidance. Finasteride‐treated rats showed significantly lower scores compared to controls (panel F, two‐tailed unpaired *t*‐test, *t* = 2.12, df = 18, *p* = 0.0484), confirming their tendency to avoid novel stimuli. These results can be appreciated in the heat map (panel G) that exemplified the behaviors of one rat per condition in the arena, showing the preference for objects in vehicle‐treated animals or for the empty zone in finasteride‐treated animals. To complement these analyses, we compared behavioral parameters across treatment phases (T0 vs. T1). As reported in Table 1 we found significant interaction (time × treatment), both in the open‐field and novelty‐seeking paradigms, confirming that the behavioral alterations were the results of changes at withdrawal and were not present immediately after finasteride treatment. Altogether, these results suggest that discontinuing finasteride leads to behavioral alterations related to anxiety‐like behavior.

**TABLE 1:** Evaluation of finasteride treatment at T0 and T1 in OF, NST and EPM.

Anova table		DF	*F* (DFn, DFd)	*p* value
*Open field test T0 × T1*				
Distance travelled	Interaction	15	*F* (15, 180) = 13.15	*p* < 0.0001
	Time	5	*F* (4.078, 146.8) = 145.0	*p* < 0.0001
	Treatment	3	*F* (3, 36) = 12.27	*p* < 0.0001
Time freezing	Interaction	15	*F* (15, 180) = 1.426	*p* = 0.1391
	Time	5	*F* (3.429, 123.4) = 5.738	*p* = 0.0006
	Treatment	3	*F* (3, 36) = 3.088	*p* = 0.0392
Center time spent	Interaction	15	*F* (15, 180) = 2.314	*p* = 0.0049
	Time	5	*F* (3.435, 123.7) = 1.218	*p* = 0.3066
	Treatment	3	*F* (3, 36) = 7.268	*p* = 0.0006
*Novelty seeking test T0 × T1*				
Object 1 time spent	Interaction	15	*F* (15, 180) = 1.609	*p* = 0.0750
	Time	5	*F* (4.005, 144.2) = 6.521	*p* < 0.0001
	Treatment	3	*F* (3, 36) = 3.834	*p* = 0.0176
Object 1 time getting closer	Interaction	15	*F* (15, 180) = 1.785	*p* = 0.0396
	Time	5	*F* (4.010, 144.4) = 22.82	*p* < 0.0001
	Treatment	3	*F* (3, 36) = 0.7330	*p* = 0.5391
Object 2 time spent	Interaction	15	*F* (15, 180) = 1.191	*p* = 0.2823
	Time	5	*F* (3.622, 130.4) = 0.9333	*p* = 0.4400
	Treatment	3	*F* (3, 36) = 1.888	*p* = 0.1491
Object 2 time getting closer	Interaction	15	*F* (15, 180) = 1.555	*p* = 0.0905
	Time	5	*F* (3.912, 140.8) = 26.28	*p* < 0.0001
	Treatment	3	*F* (3, 36) = 1.224	*p* = 0.3149
*Elevated plus maze test T0 × T1*				
Open arms entries	Interaction	1	*F* (1, 17) = 0.079	*p* = 0.7825
	Time	1	*F* (1, 17) = 12.290	*p* = 0.0027
	Treatment	1	*F* (1, 17) = 0.314	*p* = 0.5828
Closed arms entries	Interaction	1	*F* (1, 17) = 0.1854	*p* = 0.6721
	Time	1	*F* (1, 17) = 17.22	*p* = 0.0007
	Treatment	1	*F* (1, 17) = 0.1498	*p* = 0.7035
Open arms time	Interaction	1	*F* (1, 16) = 0.020	*p* = 0.8883
	Time	1	*F* (1, 16) = 7.820	*p* = 0.0129
	Treatment	1	*F* (1, 16) = 0.284	*p* = .6015
Open arms mean speed	Interaction	1	*F* (1, 17) = 0.8904	*p* = 0.3586
	Time	1	*F* (1, 17) = 0.8941	*p* = 0.3576
	Treatment	1	*F* (1, 17) = 0.8726	*p* = 0.3633
Max speed	Interaction	1	*F* (1, 17) = 0.123	*p* = 0.7302
	Time	1	*F* (1, 17) = 1.209	*p* = 0.2869
	Treatment	1	*F* (1, 17) = 0.381	*p* = 0.5455

**FIGURE 7:**
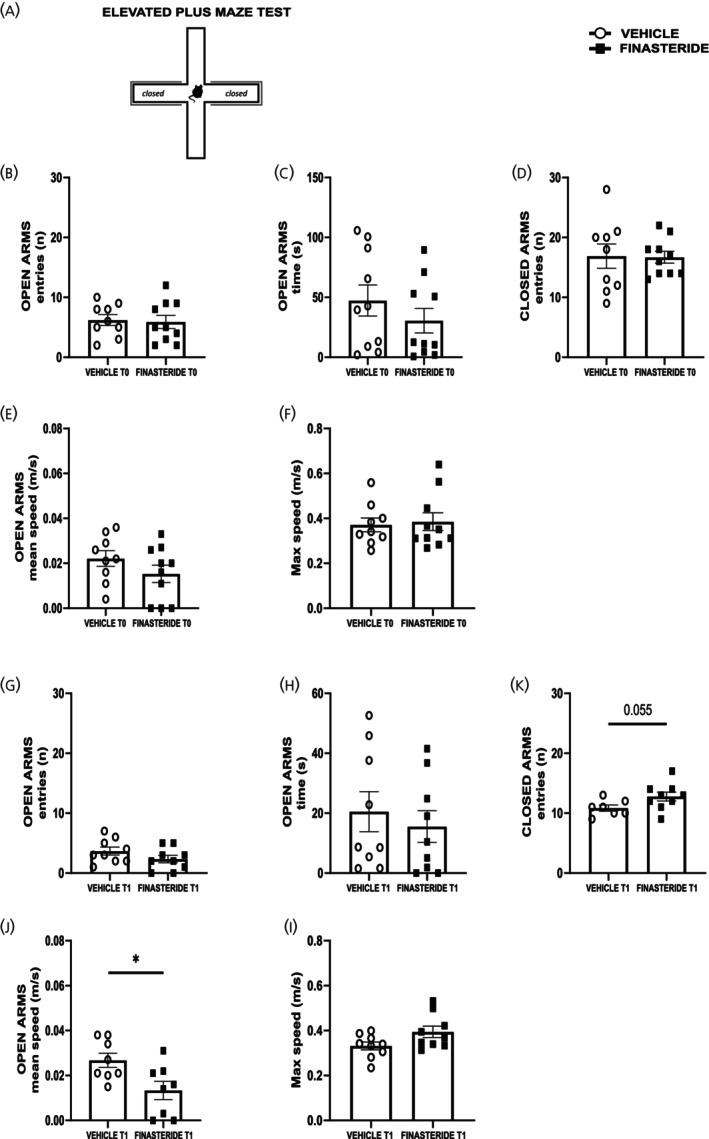
Effects of subchronic finasteride administration and its discontinuation on locomotor activity related to anxiety‐like behavioral state. (A) Schematic representation of elevated plus maze test (EPM); (B) numbers of entries in the open arms by finasteride‐treated rats compared to control vehicle rats after finasteride subchronic treatment during 5 min of EPM test (*n* = 9 (vehicle), *n* = 10 (finasteride)); (C) time spent in the open arms by finasteride‐treated rats compared to control vehicle rats after finasteride subchronic treatment during 5 min of EPM test (*n* = 10 (vehicle), *n* = 10 (finasteride)); (D) numbers of entries in the closed arms by finasteride‐treated rats compared to control vehicle rats after finasteride subchronic treatment during 5 min of EPM test (*n* = 9 (vehicle), *n* = 10 (finasteride)); (E) mean speed of vehicle‐ and finasteride‐treated rats after subchronic treatment in the open arms during 5 min of EPM test (*n* = 9 (vehicle), *n* = 10 (finasteride)); (F) max speed of vehicle‐ and finasteride‐treated rats after subchronic treatment in the EPM device during 5 min of the test (*n* = 9 (vehicle), *n* = 10 (finasteride)); (G) numbers of entries in the open arms by finasteride‐treated rats compared to control vehicle rats after finasteride discontinuation during 5 min of EPM test (*n* = 9 (vehicle), *n* = 9 (finasteride)); (H) time spent in the open arms by finasteride‐treated rats compared to control vehicle rats after finasteride discontinuation during 5 min of EPM test (*n* = 9 (vehicle), *n* = 9 (finasteride)); (K) numbers of entries in the closed arms by finasteride‐treated rats compared to control vehicle rats after finasteride discontinuation during 5 min of EPM test (*n* = 7 (vehicle), *n* = 9 (finasteride)); (J) mean speed of vehicle‐ and finasteride‐treated rats after finasteride discontinuation in the open arms during 5 min of EPM test (*n* = 8 (vehicle), *n* = 8 (finasteride)); (I) max speed of vehicle‐ and finasteride‐treated rats after finasteride discontinuation in the EPM device during 5 min of the test (*n* = 9 (vehicle), *n* = 9 (finasteride)). *p < 0.05. Values are expressed as mean ± SEM.

Based on these findings, we evaluated a distinct cohort of finasteride‐treated and control animals using the EPM (Figure 7A), a well‐established paradigm for assessing anxiety‐like behaviors in rodents. Consistent with the behavioral parameters analyzed previously, no significant alterations were observed at the baseline timepoint (T0) for either open (Figure 7B, two‐tailed Mann–Whitney test, *U* = 41, *p* = 0.7324 and C, two‐tailed Mann–Whitney test, *U* = 37, *p* = 0.3527) or closed arm exploration (panels D, two‐tailed Mann–Whitney test, *U* = 42, *p* = 0.8249) and locomotor‐related parameters (Figure 7E, two‐tailed unpaired *t*‐test, *t* = 1.30, df = 17, *p* = 0.2116 and F, two‐tailed unpaired *t*‐test, *t* = 0.28, df = 17, *p* = 0.7833).

In contrast, at time point T1, while we found no differences in entries and time spent in the open arms (panel G, two‐tailed Mann–Whitney test, *U* = 26, *p* = 0.1970 and panel H, two‐tailed Mann–Whitney test, *U* = 33, *p* = 0.5289), finasteride‐treated rats showed a trend toward more entries to the closed arms (Figure 7K, two‐tailed Mann–Whitney test, *U* = 14, *p* = 0.055), suggesting a preference to less anxiogenic component of the maze. Additionally, while finasteride‐treated animals exhibited a tendency toward higher overall locomotor velocity in the maze (Figure 7I, two‐tailed unpaired *t*‐test, *t* = 2.02, df = 16, *p* = 0.0606), consistent with the hyperlocomotor phenotype observed in alternative behavioral contexts, these subjects approached the open arms with significantly reduced mean velocity relative to control animals (Figure 7J, two‐tailed unpaired *t*‐test, *t* = 2.61, df = 14, *p* = 0.0206).

In addition, as reported in Table 1, for all EPM parameters here considered, we did not find significant interaction (time × treatment). Collectively, these results demonstrate that although finasteride has limited behavioral effects after chronic treatment, the drug suspension leads to persistent behavioral alterations characterized by increased locomotion, increased anxiety‐like behavior, and marked avoidance of novel stimuli.

## 4 DISCUSSION

Our findings demonstrate that subchronic finasteride treatment in adult male rats induces distinct time‐dependent behavioral changes. While the immediate effects observed after 20 days of treatment (i.e., T0) were modest, significant behavioral alterations became evident 1 month after drug discontinuation (i.e., T1). These withdrawal effects included pronounced hyperactivity, reduced center exploration in the open field, and marked avoidance of novel stimuli, collectively indicating an anxiety‐like phenotype. These results differ substantially from those reported by^19^ and,^25^ who observed significant anxiety‐ and depression‐like behaviors immediately following finasteride administration, albeit only at substantially higher doses (30 and 100 mg/kg compared to our 3 mg/kg). Notably, despite the use of a much lower dose, prolonged treatment duration was sufficient to induce persistent behavioral alterations that emerged at the drug withdrawal. This suggests that even at clinically relevant doses, chronic finasteride exposure may lead to enduring neuroadaptations that only become behaviorally evident following treatment cessation, a finding with important implications for understanding the PFS observed in patients.

The behavioral effects appear to differ markedly following drug withdrawal. Notably, 1 month after treatment suspension (i.e., a timeframe that mirrors the condition of PFS patients), finasteride‐treated animals exhibited a significant increase in locomotor activity, a marked reduction in the immobility time, and spent significantly less time in the center of the open field. Collectively, these findings are indicative of an anxiety‐like behavioral phenotype. This aligns with clinical observations in PFS patients, where anxiety is one of the most frequently reported symptoms following finasteride withdrawal.^8^, ^26^, ^27^, ^28^

Our study also revealed that drug withdrawal induced marked avoidance of novel stimuli, as indicated by reduced object exploration and increased time spent in the empty corners of the test arena. This previously undocumented behavior is commonly associated with anxiety‐like states in rodents^29^ and is consistent with findings in clinical populations, where anxiety correlates with decreased novelty‐seeking behavior.^30^ While^19^ reported deficits in spatial memory in the novel object location test following finasteride treatment, our paradigm specifically focuses on exploratory behavior and novelty avoidance rather than cognitive aspects. Nonetheless, cognitive functions might also be impaired in our experimental model. It will therefore be important to explore this aspect. For instance, it has been reported that dopamine transporter knock‐out (DAT KO) rats exhibit increased locomotion associated with cognitive alterations,^31^ suggesting that a similar interaction could also be possible in our experimental model. Indeed, our findings complement previous observations by showing that finasteride withdrawal not only affects cognitive function but also fundamentally induces long‐term behavioral changes. In addition, decreased novelty‐seeking behavior may also be associated with a decrease in hippocampal neurogenesis.^32^, ^33^ In agreement with behavioral data here we demonstrated that reported, in the same experimental model, finasteride withdrawal induced a decrease in neurogenesis (i.e., number of pH 3 immunoreactive cells) in the subgranular zone of the dentate gyrus.^17^

It is also interesting to note that recent RNA sequencing analysis in rats treated with finasteride showed molecular alterations not only after treatment but also at the drug withdrawal period, in both the hypothalamus and hippocampus.^21^ While hypothalamic functions are not directly related to the behavioral tasks assessed in this study, the hippocampus is likely involved in some of the observed alterations. Specifically, the hippocampus plays a key role in spatial novelty detection and contextual processing, which can influence novelty‐seeking behavior.^34^ The alteration identified in these sequencing studies further supports the idea that finasteride administration impacts not only behavior but also underlying neural mechanisms. Further insights focusing on the molecular alterations leading to changes in behavior will be explored in future experiments.

The anxiety‐like behavior observed in this study is likely associated with changes in neuroactive steroid levels.^35^, ^36^, ^37^ Indeed, we have demonstrated that both PFS patients,^15^, ^38^, ^39^ and the experimental model used here^22^ reported a general decline in neuroactive steroid levels. In particular, we observed that finasteride treatment not only affected, as expected, the levels of 5α‐reduced metabolites of PROG and T but also related neuroactive steroids, suggesting a general impairment of steroidogenesis/neurosteroidogenesis. Interestingly, among all these altered levels of neuroactive steroids, those of allopregnanolone were significantly decreased.^15^, ^22^ This neuroactive steroid is a potent ligand of GABA‐A receptors,^40^ and GABAergic system dysregulation has been associated with anxiety‐like behaviors.^41^ Our previous observations obtained in the experimental model utilized in this study have also indicated altered cerebral expression levels of some GABA‐A subunits,^22^ suggesting that the behavioral effects observed at finasteride withdrawal may be related to these mechanisms.

Collectively, our findings provide compelling evidence that while finasteride has limited immediate behavioral effects during chronic treatment, drug discontinuation leads to persistent behavioral changes characterized by hyperactivity and increased anxiety‐like behaviors. These results, besides showing a picture of increased vulnerability, align with clinical reports in PFS and highlight the importance of monitoring patients not only during treatment but also following discontinuation of finasteride therapy. Despite the widespread use of finasteride for treating AGA and BPH, the long‐term neuropsychiatric consequences of this drug remain largely unknown, particularly after treatment cessation. Our findings underscore the need for further research into the neurobiological mechanisms underlying these persistent post‐discontinuation effects, particularly focusing on the role of neuroactive steroids and GABAergic function.

## AUTHOR CONTRIBUTIONS

Lucia Cioffi conceptualized the experiments, performed animal treatment and behavioral analyses, wrote the original draft and revised the text; Silvia Diviccar conceptualized the experiments, performed animal treatment and behavioral analyses, wrote the original draft and revised the text; Gabriela Chrostek conceptualized the experiments, performed animal treatment and behavioral analyses; Francesco Paolo Ulloa Severino wrote the original draft and revised the text; Silvia Giatti conceptualized the experiments, performed animal treatment and behavioral analyses, wrote the original draft and revised the text; Diego Scheggia conceptualized the experiments, wrote the original draft and revised the text; Roberto Cosimo Melcangi general supervision; conceptualized the experiments, wrote the original draft and revised the text.

## FUNDING INFORMATION

This research was funded by grants from MUR Progetto Eccellenza (2023–2027) to the Department of Pharmacological and Biomolecular Sciences “Rodolfo Paoletti” Università degli Studi di Milano and the Post‐Finasteride Syndrome Foundation to Roberto Cosimo Melcangi.

## CONFLICT OF INTEREST STATEMENT

None.

## Data Availability

The data that support the findings of this study are available on request from the corresponding author. The data are not publicly available due to privacy or ethical restrictions.
